# Diet, feeding, and niche overlap of west coast steenbras (*Lithognathus aureti*) and silver kob (*Argyrosomus inodorus*) in the northern Benguela

**DOI:** 10.1111/jfb.15914

**Published:** 2024-08-23

**Authors:** Arariky S. Shikongo, Margit R. Wilhelm

**Affiliations:** ^1^ Department of Fisheries and Ocean Sciences, Sam Nujoma Campus University of Namibia Henties Bay Namibia

**Keywords:** diet composition, feeding habits, index of relative importance, niche overlap, stomach content analysis

## Abstract

In this study, we described and compared the diet, monthly feeding intensity, and condition of west coast steenbras (*Lithognathus aureti*) and silver kob (*Argyrosomus inodorus*) caught at a unique habitat in the northern Benguela. Stomach contents of 179 west coast steenbras and 114 silver kob caught from October 2020 to September 2022 were investigated. The peak in feeding intensity of west coast steenbras appeared to be opportunistic during winter and summer periods depending on food availability. The fish condition, however, peaked at the beginning (October) and at the end (April) of the austral summer spawning period, with the hepatosomatic index (HSI) at 1.5% and the condition factor (CF) at 0.022%. Seven prey taxa were found in the diet of west coast steenbras (bivalves, bony fishes, other mollusks, algae, crustaceans, cnidaria, and polychaetas) and six taxa in the diet of silver kob (bivalves, crustaceans, bony fishes, algae, starfish, and zooplankton), indicating generalist feeding behavior in both the species. The bivalves were the most important prey items in the diet of west coast steenbras (95.9% index of relative importance [IRI]). The most important prey items in the diet of silver kob were crustaceans (83.1% IRI) and bony fishes (16.0% IRI). Crustaceans were most important in the diet of small‐to‐medium‐sized silver kob, whereas bony fishes were most important in the diet of larger silver kob (>75 cm), with significant differences of IRI% by size class. Schoener's index of niche overlap indicated a relatively low overall niche overlap (0.11) between west coast steenbras and silver kob. This allows them to coexist as their feeding habits allow them to occupy unique niches in the coastal reef and sandy habitat and reduce competition for resources.

## 1 INTRODUCTION

The Namibian coast is renowned for its angling opportunities, particularly in the central and northern regions, and the 1996/1997 season brought a direct annual expenditure (by 8800 anglers) of N$ 29.7 million (USD 1.7 million, Kirchner et al., 2000; Stage & Kirchner, 2005). In a more recent economic study, the estimate ranged between N$137 million and N$1.03 billion of total direct annual expenditures, depending on how angler numbers were estimated (Khan, 2023). Although the diversity of linefish species is low, their abundance has been high with evidence of a long history of the harvesting of marine fish by linefishing, the main species harvested being silver kob (*Argyrosomus inodorus*) and west coast steenbras (*Lithognathus aureti*) (Holtzhausen et al., 2001).

West coast steenbras belongs to the Sparidae or sea bream family (Smith & Smith, 1986). Although its range stretches from Rio Longa (10.234° S, 13.483° E), Angola to Cape Town, South Africa (34.21° S, 18.64° E) (Mann et al., 2014), Namibia holds a distinct, closed population at Meob Bay (24.77° S, 14.76° E to 24.45° S, 14.60° E, van der Bank & Holtzhausen, 1999). Tagging studies and genetic analysis revealed this population's separation from a northern Namibian one (van der Bank & Holtzhausen, 1999). West coast steenbras exhibits protandrous hermaphroditism, with males typically changing sex after reaching maturity (Holtzhausen & Kirchner, 2001). Spawning occurs in the austral summer (October–February) with a peak in December–February (Holtzhausen, 1999). Age at 50% maturity varies between the northern and southern populations, ranging between 4.8 and 6.0 years for males and between 7.2 and 9.7 years for females. Eggs and larvae likely drift northward with the Benguela Current after spawning in the surf zone (Holtzhausen, 1999). Previous research on the diet of west coast steenbras focused on the southern population, finding black mussels (*Choromytilus merdionalis*) and brown mussels (*Perna perna*) as their primary prey (Holtzhausen & Kirchner, 2001). They also reported a higher percentage of empty stomachs in the southern population compared to the northern population, suggesting potential food availability differences influencing growth rates. To our knowledge, no further diet studies on west coast steenbras in Namibia have been published.

**FIGURE 1:**
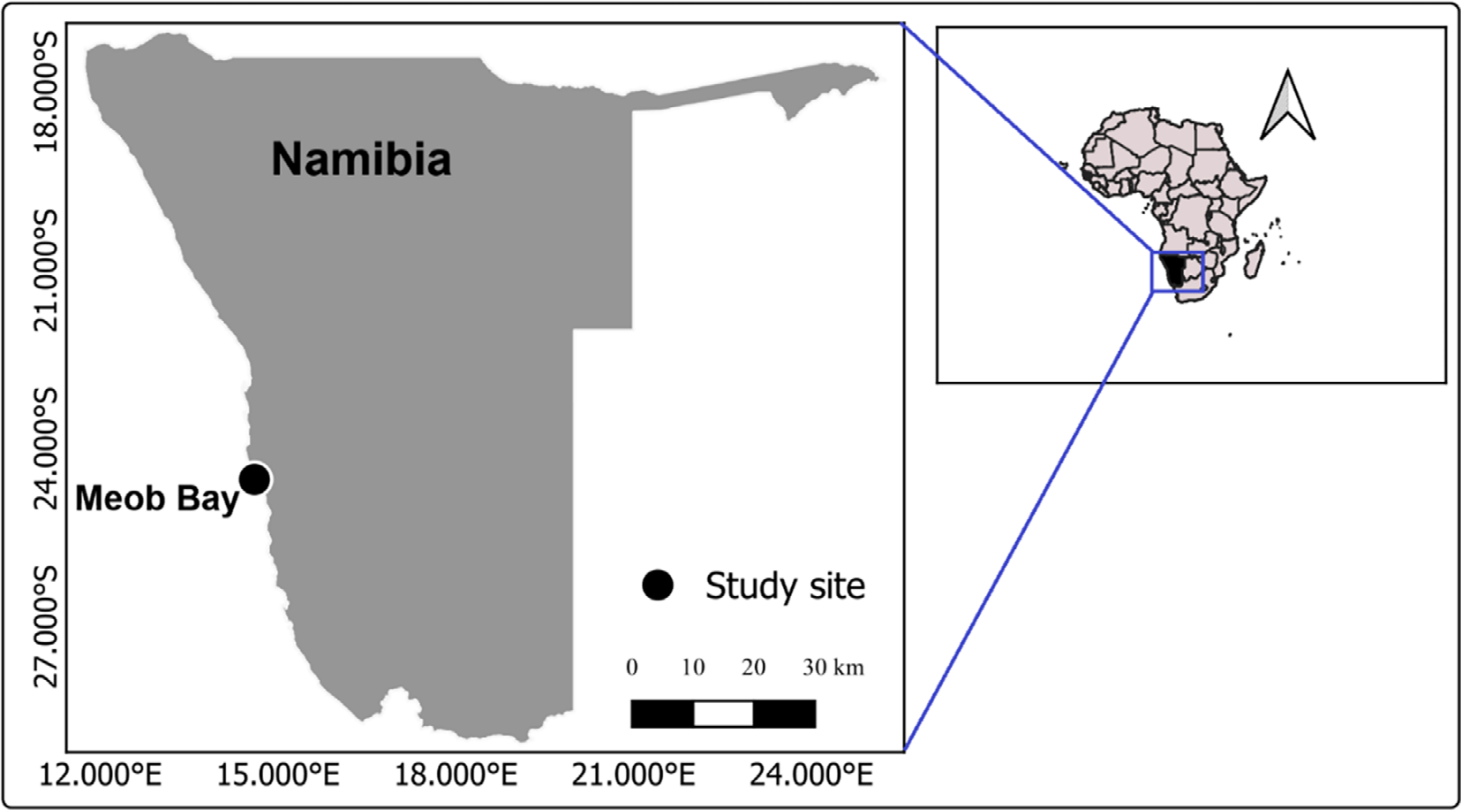
Map showing the location of study site at Meob Bay, southern Namibia.

Silver kob belongs to the Sciaenidae family and is found along the southern African coast, with one distinct stock in Namibia (Kirchner & Holtzhausen, 2001) and potentially three in South Africa (Griffiths, 1997). Genetic studies support this distinction between the Namibian and South African populations (Henriques et al., 2014). Namibian silver kob undertake seasonal migrations (Kirchner & Holtzhausen, 2001). Adults migrate south for spawning (October–March) to Sandwich Harbour and Meob Bay, with larvae then drifting northward to nursery grounds (Figure 1). Juveniles eventually move north to Skeleton Coast National Park (SCNP, 17‐20°S) waters as they mature (around 2 years old). This migratory pattern highlights the importance of understanding stock dynamics for effective management across their range.

Silver kob undertake seasonal migrations, with adults leaving the SCNP for spawning grounds near Meob Bay during summer (Kirchner & Holtzhausen, 2001). This migration makes them vulnerable to overfishing, particularly during spawning aggregations (Kirchner, 1998). To address this, closed areas and seasons were implemented, restricting fishing near the shores and by boat between Pelican Point and Sandwich Harbour from January to March. Management strategies include a bag limit of 10 fish per day and a size restriction of 40–70 cm (Kirchner, 1998). However, the effectiveness of these regulations, established in the late 1990s, has not been reassessed. Understanding the diet of silver kob, which is only known to include smaller fish, crustaceans, and squid (Bianchi et al., 1999), is crucial for future management plans. Studying their diet can help create regulations that consider the broader ecosystem impacts of silver kob fishing.

Knowledge of fish diets is crucial for understanding their ecological roles within food webs and energy flow (Ainsworth et al., 2010). This information is incorporated into ecological models to represent interactions between species and top predators (e.g., Christensen, 1995). Furthermore, dietary studies help to estimate trophic levels, which are useful for assessing the impact of fishing on marine ecosystems (Pauly et al., 2000). For instance, overfishing of prey species can lead to declines in top predator populations (Furness & Tasker, 2000). In Namibia, unregulated fishing has depleted prey like sardines and anchovies, potentially impacting predators such as Cape fur seals (Erasmus et al., 2021).

Stomach content analysis is a common method to investigate fish diets, revealing the types and quantities of consumed prey (Ainsworth et al., 2010). These analyses can be used to explore how diet changes with age, size, competition, spatial factors, and seasonal variations (e.g., Crespin De Billy et al., 2000). Although Namibian linefish research has seen advancements (Holtzhausen, 1999; Kirchner & Voges, 1999), there is still a lack of recent studies, specifically on the diet and feeding habits of west coast steenbras and silver kob, particularly in southern Namibia. Understanding these aspects is essential for effective conservation strategies (Gusha et al., 2024), especially because west coast steenbras has been identified as a species vulnerable to climate change (Engelhard et al., 2024).

This study aims to address this gap in knowledge by investigating and comparing the diets of west coast steenbras and silver kob in Meob Bay (southern Namibia) from 2020 to 2022. By analysing their dietary overlap, the study seeks to identify potential ecological interactions between these species, particularly regarding competition for resources. The decision to study them together was based on their co‐occurrence in Meob Bay, with silver kob migrating there for spawning. This raised questions about potential competition, which this research aims to address by examining their diets simultaneously.

## 2 MATERIALS AND METHODS

The care and use of animals complied with Namibian animal welfare laws, guidelines, and policies as approved by National Commission for Research Science and Technology (NCRST), Namibia, research permit reference number: RCIV00022018.

### 2.1 Study area

The study was conducted at Meob Bay, a marine reserve area that lies between Langewand (24.77° S, 14.76° E) and Witklip (24.45° S, 14.60° E) in the Namib Naukluft Park (NNP), in southern Namibia, about 173 km south of Walvis Bay and 243 km north of Lüderitz (Figure 1). The 50‐km‐long coastline in this area has ecological features that set it apart from the rest of the Namibian coast. Cooler waters influenced by the Lüderitz upwelling cell and a well‐defined biological boundary near the area (Agenbach & Shannon, 1988) contribute to this habitat differentiation. Furthermore, Meob Bay harbors an isolated population of the giant bivalve mollusk, *Panopea glycymeris*, not found elsewhere northwards of Sylvia Hill (25.1 °), thus this area representing the northernmost limit of the distribution of many species (Currie et al., 2008). This area also provides a rich feeding ground for west coast steenbras. The abundance of white mussels (*Donax serra*), a primary food source for west coast steenbras, thrives in the surf zone (Holtzhausen, 1999). Additionally, several rocky shore mussel species, like brown mussels and black mussels, also favored by west coast steenbras, are present (van der Bank & Holtzhausen, 1999). Meob Bay's unique ecosystem and its importance to fish populations highlight the value of its protected status.

### 2.2 Sampling

A total of 179 west coast steenbras (51 females, 53 males, and 75 hermaphrodites), with fork lengths (FL) ranging between 26.3 and 67.7 cm, and 114 silver kob (56 females and 58 males), with total lengths (TL) ranging between 33.7 and 81.9 cm, were sampled for diet composition analysis from October 2020 to September 2022. The individual fish lengths (both TL and FL in the case of west coast steenbras) were measured to the nearest millimeter using a measuring board, and the fish were weighed in terms of total body weight and gutted body weight to the nearest 10 g using an electronic balance. The gonads, liver, and stomach were removed from the fish, and the full gut, liver, and gonads were individually weighed to the nearest 0.1 g. Sex was assigned to each fish based on macroscopic characteristics of their gonads.

The stomachs were cut open and the content was removed; prey items were identified and separately weighed (to the nearest 0.1 g) and counted when possible. The examined food items included those found in the alimentary canal as far as the first bend of the gut. Each prey item found in the diet of the fish was identified to the lowest possible taxon according to broad taxonomic groups where possible, using the Field Guide to the Living Marine Resources of Namibia (Bianchi et al., 1999) and a Guide to the Marine Life of Southern Africa (Branch et al., 2007). Any remains of algae were treated as a count of one and were weighed separately.

To estimate the count of individual prey items of mussels or bivalves that were broken and could not be counted in fish stomachs, intact empty mussel shells (*C. meridionalis*) were sampled and weighed (at Meob Bay). An average weight per individual was calculated for mussels. This average individual weight (*I*_*w*_) was then used for count conversions:

(1) $$N=W/I_{w}$$

where *N* is the number of prey items, *W* is the measured weight of the mussel remains in the fish stomach. For crustaceans, it was also assumed that the mean weight of each individual prey item was constant based on samples that were intact and could be counted.

### 2.3 Data analysis

To investigate seasonal feeding intensity, a stomach fullness index (SFI) in percentages (Man & Hodgkiss, 1977) was calculated for each month as follows:

(2) $$\mathrm{SFI}=\frac{\text{Stomach content mass}}{\text{Eviscerated fish mass}}\times 100$$

where empty stomachs were counted as having 0 content mass.

The proportion of empty stomachs and the proportion of non‐empty stomachs, with only unidentified digested material (i.e., nearly empty stomachs), were also calculated for each month. For seasonal context, the following condition indices were also calculated per month: The hepatosomatic index (HSI) was calculated according to Pardoe et al. (2008):

(3) $$\mathrm{HSI}=\frac{\mathrm{Wl}\;}{\text{We}}\times 100x$$

where Wl = liver weight (in grams); We = eviscerated fish weight (gutted weight) (in grams).

The condition factor (CF) was calculated using the following equation (Weatherly, 1972):

(4) $$\mathrm{CF}=\frac{W}{L^{3}}\times 100x$$

where *W* = the total fish wet weight (in grams) and *L* = the length of the fish (TL for silver kob and FL for west coast steenbras) in millimeters. HSI and CF were pooled for all sexes as no visual differences in peaks for the sexes were observed (Shikongo, 2024, see also Figures S1 and S2).

Numerous indices have been used to describe the importance of different prey items in the fish diet. The main food items were identified using the index of relative importance (IRI) (Pinkas et al., 1971). The IRI was calculated in a manner similar to that of Konan et al. (2011):

(5) $$\text{Percentage frequency of occurrence}\;\left(\mathrm{FO}\%\right)=\frac{N_{\mathrm{gi}}}{N_{t}}\times 100$$

where *N*_*gi*_ = the number of guts that contained prey item *i*, and *N*_*gt*_ = the total number of non‐empty guts. % F was calculated for unidentifiable organic material, but then non‐empty stomachs were only those that had identifiable prey items in them. (FO% adds up to >100% for all prey items as one fish can have more than one prey item present in its stomach.)

(6) $$\text{Numerical percentage of abundance}\;\left(N\%\right)=\frac{N_{i}}{N_{t}}\times 100$$

where *N*_*i*_ = the total number of prey item *i*, and *N*_*t*_ = the total number of all prey items for all samples (excluding stomachs with only digested unidentifiable material) combined.

(7) $$\text{Weight percentage}\;\left(W\%\right)=\frac{W_{i}}{W_{t}}\times 100$$

where *W*_*i*_ = the total weight of prey item *i*, and *W*_*t*_ = total weight of all prey items for all samples combined (excluding stomachs with only digested unidentifiable material).

The IRI combines FO%, N%, and weight W%:

(8) $$\mathrm{IRI}=\mathrm{FO}\%\left(N\%+W\%\right)$$

Seasonal variation in diet was investigated by comparing the IRI% of all fish sampled in the austral warm season (October–March) and cold season (April–September). To test for differences in IRI% between length groups, sexes, and seasons, a non‐parametric Kruskal–Wallis test was performed for each food group.

Schoener's index (*D*) for niche overlap of the diet consumed by west coast steenbras was calculated according to Schoener (1968):

(9) $$\;D=1-\frac{1}{2}\sum_{i}\left(\;|P_{\mathrm{ij}}-P_{\mathrm{ik}}\right)$$

where, *P*_*i,j*_ and *P*_*i,k*_ are relative proportions of prey item *i* by wet weight (Equation 7) in the diets of species *j* (silver kob) and species *k* (west coast steenbras).

All statistical analyses were performed using Microsoft Excel or Minitab version 17.0 (https://www.minitab.com).

## 3 RESULTS

**FIGURE 2:**
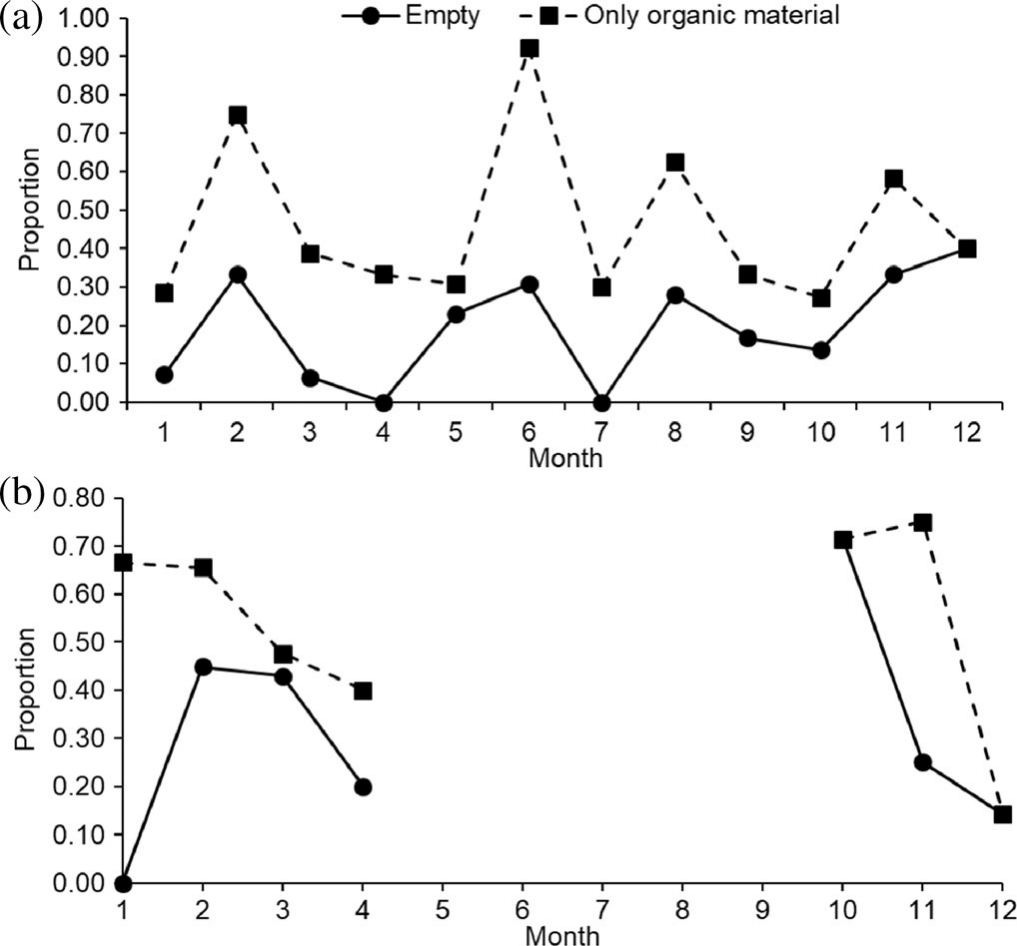
Proportion of fish with empty stomachs (circles) and those with only unidentifiable digested organic material in their stomachs, that is, nearly empty stomachs (squares) of (a) west coast steenbras *Lithognathus aureti* and (b) silver kob *Argyrosomus inodorus* by month at Meob Bay in southern Namibia. The lines are stacked to give the total proportion.

**FIGURE 3:**
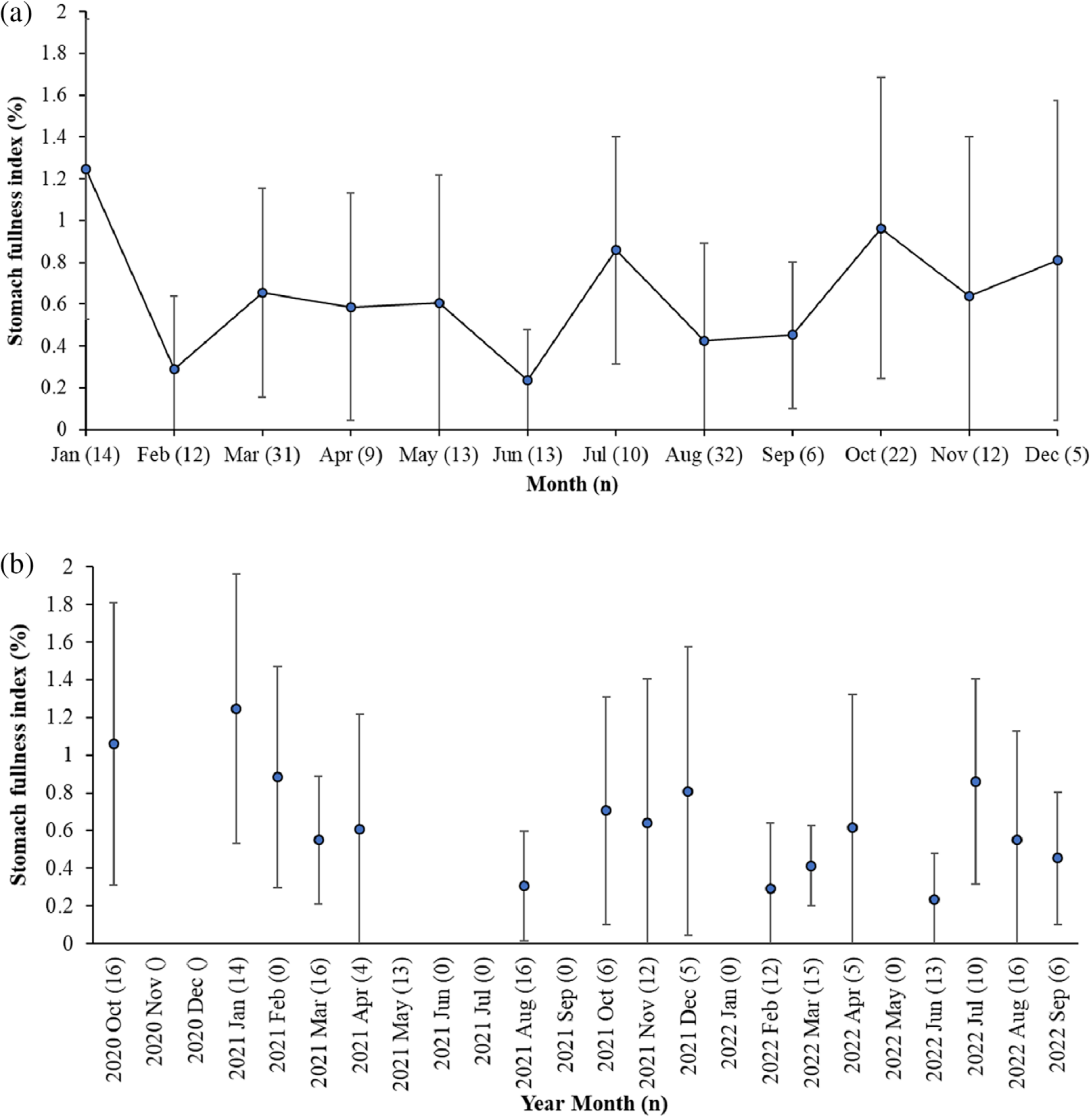
Mean monthly stomach fullness index (%) ± SD of *n* = 179 west coast steenbras *Lithognathus aureti* sampled at Meob Bay, Namibia, from October 2020 to September 2022 (a) pooled for all months and (b) separated by month and year. Sample sizes for each month are indicated in parentheses.

**FIGURE 4:**
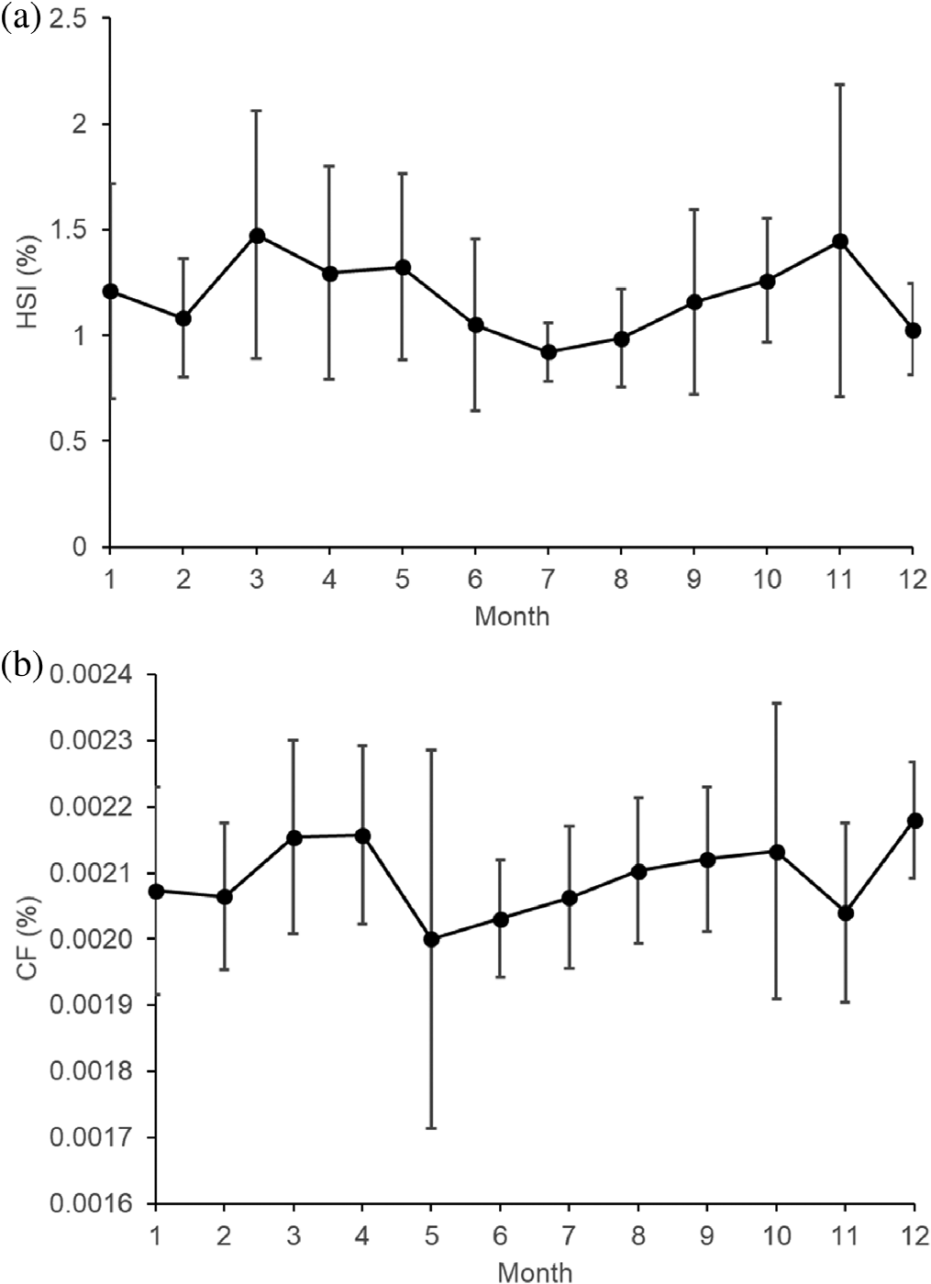
Monthly changes in (a) mean hepatosomatic index and (b) mean condition factor ± SD of west coast steenbras *Lithognathus aureti* from Meob Bay, southern Namibia (2020–2022).

Out of the 179 sampled west coast steenbras, 33 (18.4%) had empty stomachs and 51 (28.5%) had only digested organic material in their stomachs, that is, nearly empty stomachs. Most west coast steenbras with empty stomachs and nearly empty stomachs were present in February, June, August, and November (Figure 2a). Out of the 114 silver kob throughout the sampling period, 46 (40.4%) had empty stomachs, and 15 (13.2%) had only digested unidentified organic material in their stomachs. Silver kob were only caught at Meob Bay during austral summer (October–April), and most silver kob had had empty stomachs or nearly empty stomachs in October (Figure 2b). The SFI of west coast steenbras was highest in January, July, and October and at its lowest in February, June, and August–September (Figure 3). The HSI of west coast steenbras and the CF had a similar trend with peaks in March or April and then October or November (Figure 4). It did not show a similar winter peak as the SFI (Figure 3).

**FIGURE 5:**
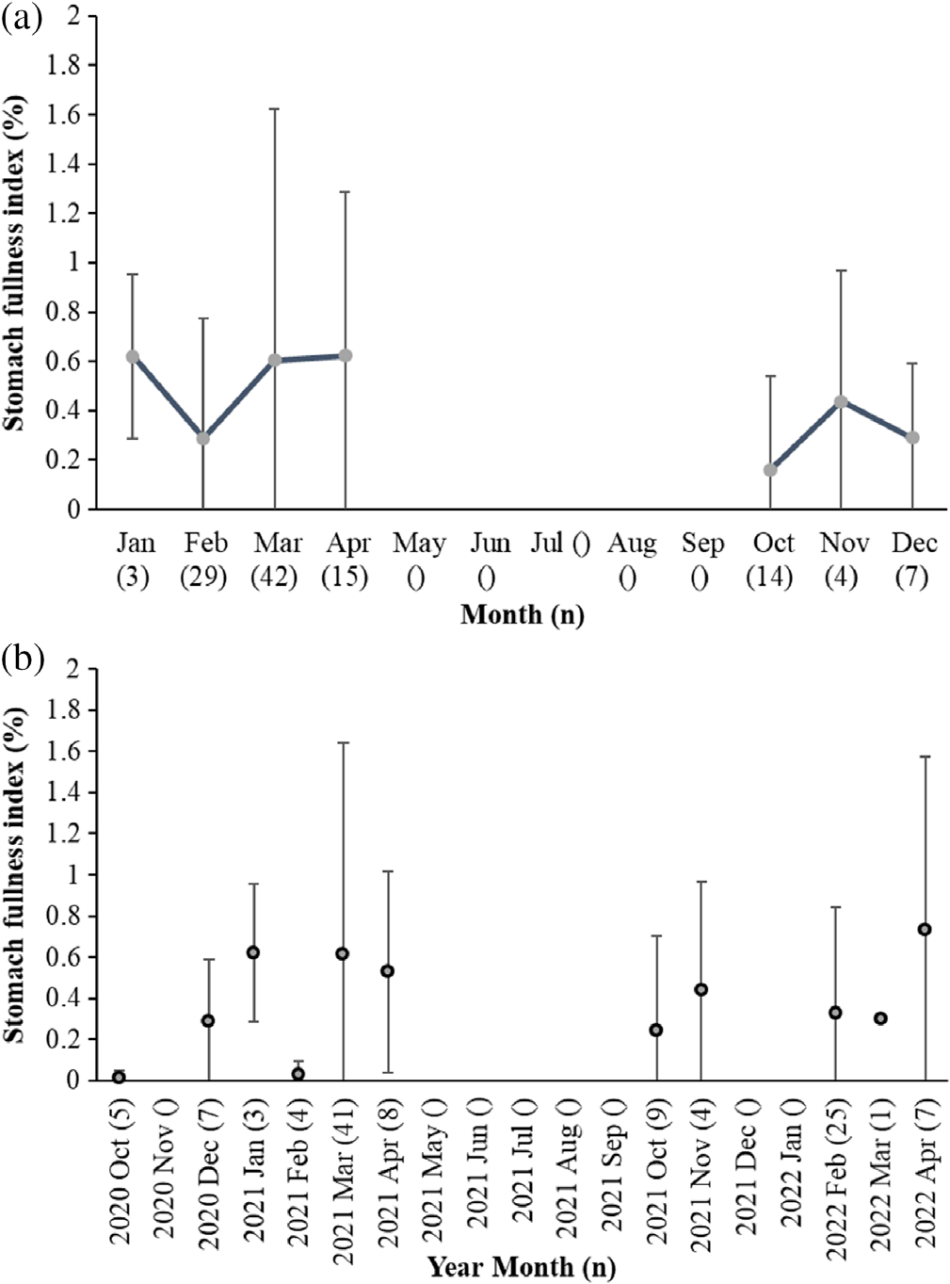
Mean monthly stomach fullness index (%) ± SD for *n* = 114 silver kob *Argyrosomus inodorus* from Meob Bay from October 2020 to April 2022 (a) pooled for all months and (b) separated by month and year. Sample sizes for each month are indicated in parentheses.

**FIGURE 6:**
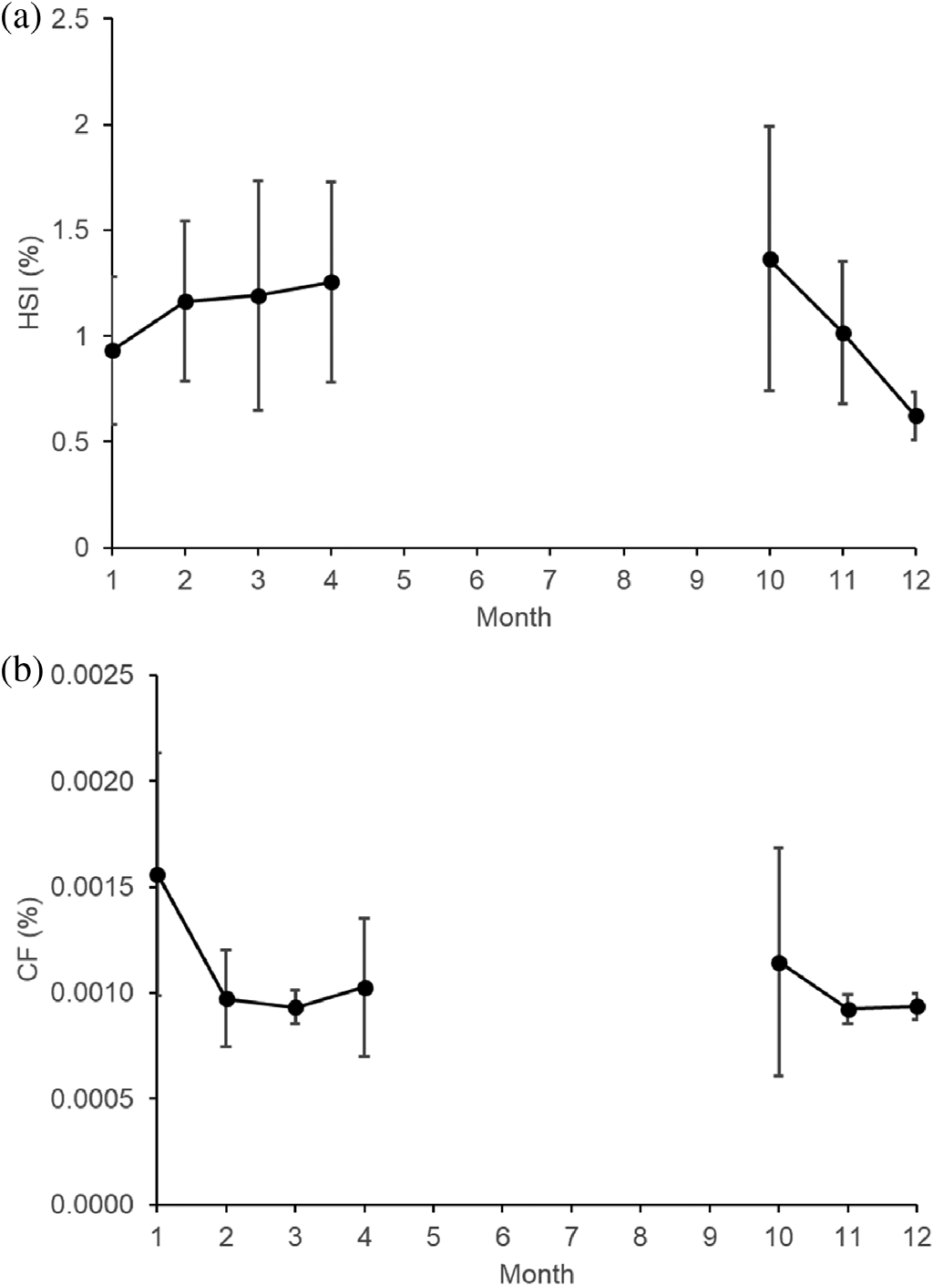
Monthly changes in (a) mean hepatosomatic index and (b) mean condition factor ± SD of silver kob *Argyrosomus inodorus* from Meob Bay, southern Namibia (2020–2022).

The SFI of silver kob was lowest in February (2021) and October (2020) (Figure 5). The HSI was similar throughout the summer period with high SDs (Figure 6a), and the CF was, on average, much lower than that of west coast steenbras and also was similar throughout the summer period (Figure 6b).

The west coast steenbras’ stomach contents comprised seven primary prey taxa, including bivalves (mainly consisting of broken shells) and other gastropods, algae, bony fishes, polychaeta (not identified to family or genus level), crustaceans (some identified to genus level), and unidentified cnidarians. Bivalves were the prey of highest relative importance of west coast steenbras, accounting for 95.93% of the total IRI, with the black mussel *C. meridionalis* being the most represented (96.36% IRI). Consumption of cnidarians and limpets was infrequent, with an IRI% of <0.05% (Table 1).

**TABLE 1:** List of prey items found in the stomachs of west coast steenbras *Lithognathus aureti* caught at Meob Bay from 2020 to 2022, with their frequency of occurrence (FO%), numerical percentage (N%), weight percentage (W%), and index of relative importance (IRI%).

Prey taxa	FO%	N%	W%	IRI%
Organic material	39.04			
Algae				
Algae—indeterminate	3.06	0.18	2.05	0.07
*Porphyra capensis*	2.04	0.13	1.28	0.03
*Ulva* spp.	17.35	2.24	12.52	2.62
Cnidaria				
Jellyfish—indeterminate	2.04	0.13	1.52	0.03
Polychaeta				
Worm—intermediate	3.06	1.56	4.01	0.17
Gastropoda				
Limpets—indeterminate	1.02	0.32	0.96	0.01
Bivalves				
*Choromytilus meridionalis*	61.22	87.27	66.30	96.36
*Donax serra*	1.02	0.19	1.60	0.02
*Perna perna*	4.08	5.11	3.77	0.37
Bivalves—indeterminate	1.02	0.06	1.44	0.02
Crustaceans				
Shrimps—indeterminate	4.08	1.73	1.28	0.13
Bony fishes				
*Sardinops ocellatus* (sardine)	4.08	0.48	2.81	0.14
Fish—indeterminate	2.04	0.59	0.44	0.02
Totals				
Organic material	39.04			
Algae	22.45	2.55	15.85	3.55
Cnidaria	2.04	0.13	1.52	0.03
Polychaeta	3.06	1.56	4.01	0.15
Limpets	1.02	0.32	0.96	0.01
Bivalves	67.35	92.63	73.12	95.93
Crustaceans	4.08	1.73	1.28	0.11
Bony fishes	6.12	1.07	3.25	0.23
Total percentage	106.1	100.0	100.0	100.0

**FIGURE 7:**
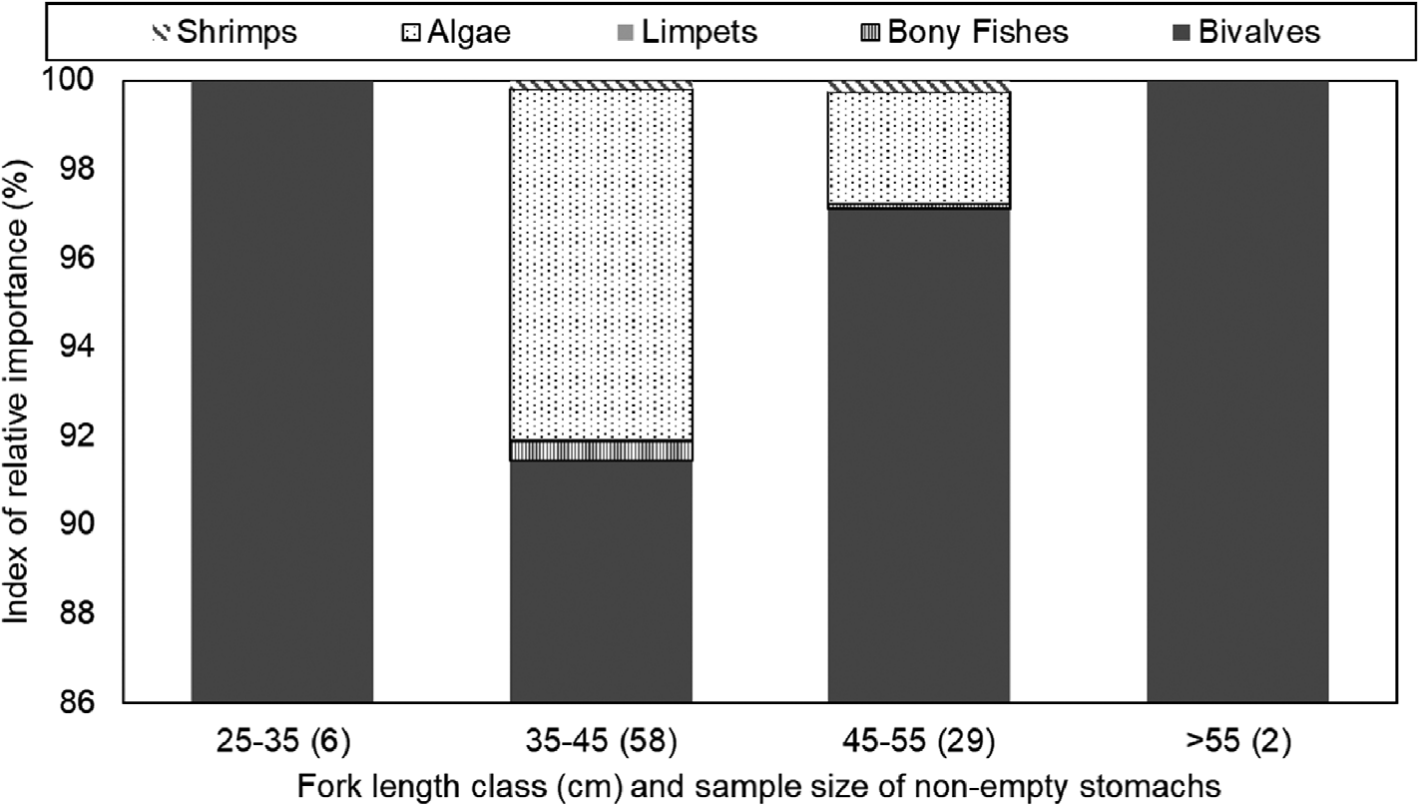
Index of relative importance (IRI%) of the main prey items consumed by west coast steenbras *Lithognathus aureti* at Meob Bay by fork length size class from 2020 to 2022.

It appeared that as the west coast steenbras grew larger, their reliance on algae in their diet decreased, and the IRI% of bivalves increased (Figure 7), but because of the small sample sizes at the small and large size classes, statistically there was no significant difference by size class (H = 6.67, *df* = 3, *p* = 0.083). The IRI% of prey taxa of west coast steenbras did not vary significantly by sex (H = 1.09, *df* = 2, *p* = 0.579) or by warm or cold season (H = 0.10, *df* = 1, *p* = 0.752).

The diet of silver kob consisted of six primary prey taxa: crustaceans (with some identified to genus level), bony fishes, bivalves (mainly consisting of broken shells), zooplankton (unidentified copepods), algae, and echinoderms (not identified to family or genus level). Crustaceans were the most significant prey group, making up 83.12% of the total IRI, followed by bony fishes at 15.96%. The main representation of crustaceans in the diet was shrimp and/or prawns, accounting for around 79% of the total IRI. Conversely, echinoderms and algae were of little importance, with an IRI of <0.1% (Table 2).

**TABLE 2:** List of prey items found in the stomachs of silver kob *Argyrosomus inodorus* caught at Meob Bay from 2020 to 2022, with their frequency of occurrence (FO%), numerical percentage (N%), weight percentage (W%), and index of relative importance (IRI%).

Prey taxa	FO%	N%	W%	IRI%
Organic material	33.33			
Algae				
*Laminaria pallida*	1.85	0.08	0.29	0.01
*Ulva* spp.	3.70	0.18	0.66	0.04
Zooplankton				
Copepods—indeterminate	7.41	0.91	0.68	0.16
Crustaceans				
*Macropetasma Africana*	44.44	71.78	53.47	77.37
Shrimps—indeterminate	7.41	10.68	7.96	1.92
Crabs—indeterminate	1.85	0.08	0.29	0.01
Bivalves				
*Choromytilus meridionalis*	5.56	6.72	5.01	0.91
Echinoderms				
Starfish—indeterminate	1.85	0.59	0.44	0.03
Bony fishes				
*Clupeidae* spp.	35.19	8.86	31.10	19.54
Fish—indeterminate	1.85	0.12	0.09	0.01
Totals				
Organic material	33.33			
Algae	5.56	0.26	0.96	0.07
Zooplankton	7.41	0.91	0.68	0.13
Crustaceans	53.70	82.54	61.72	83.12
Bivalves	5.56	6.72	5.01	0.70
Echinoderms	1.85	0.59	0.44	0.02
Bony fishes	37.04	8.98	31.19	15.96
Total percentage	111.11	100	100	100

**FIGURE 8:**
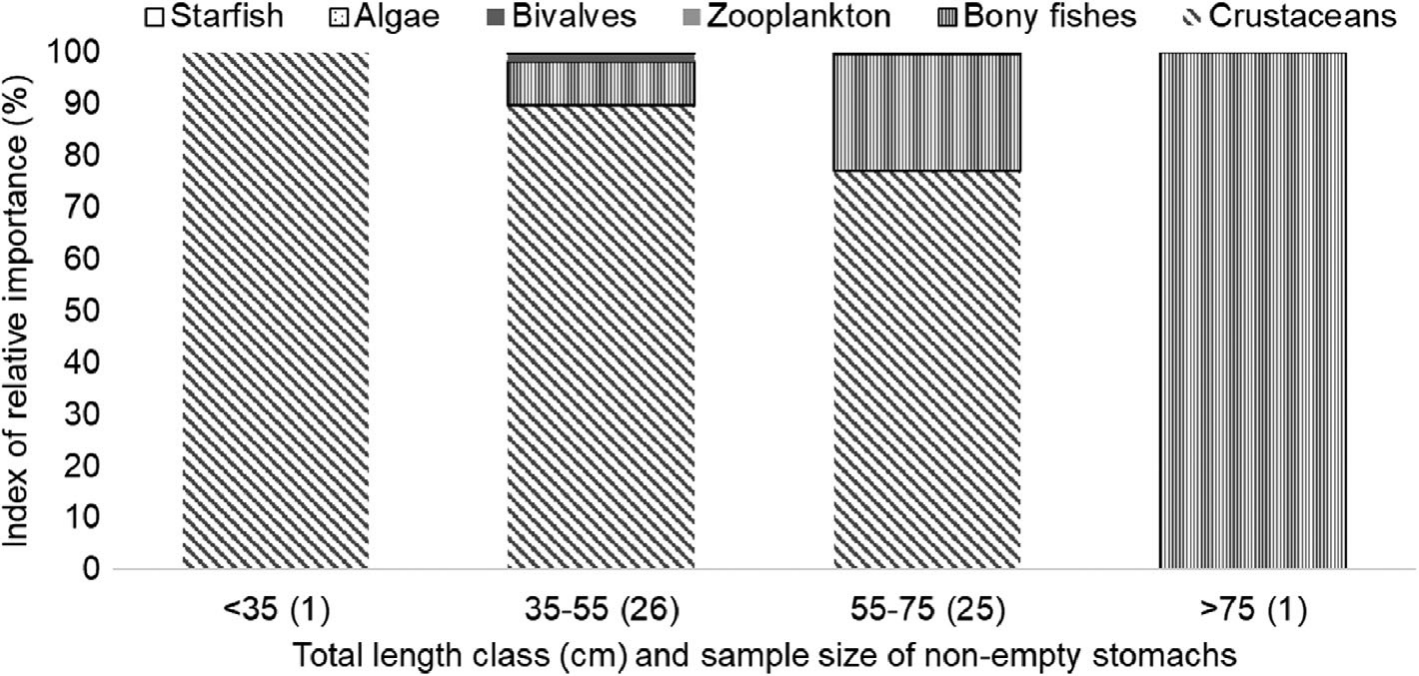
Index of relative importance (IRI%) of the main prey items consumed by silver kob *Argyrosomus inodorus* at Meob Bay by total length size class from 2020 to 2022.

The diet composition of silver kob in relation to major prey taxa showed a significant difference with size class (Figure 6; H = 9.42, *df* = 3, *p* = 0.024), but it did not vary significantly by sex (H = 0.81, *df* = 1, *p* = 0.368). The primary prey in the diet of silver kob was crustaceans, but their significance decreased with increasing size from 100% of the IRI for fish <35 cm TL, 80.3% for those between 35 and 55 cm, and 73.1% for fish between 55 and 75 cm to 100% for silver kob of the largest size class (Figure 8).

**FIGURE 9:**
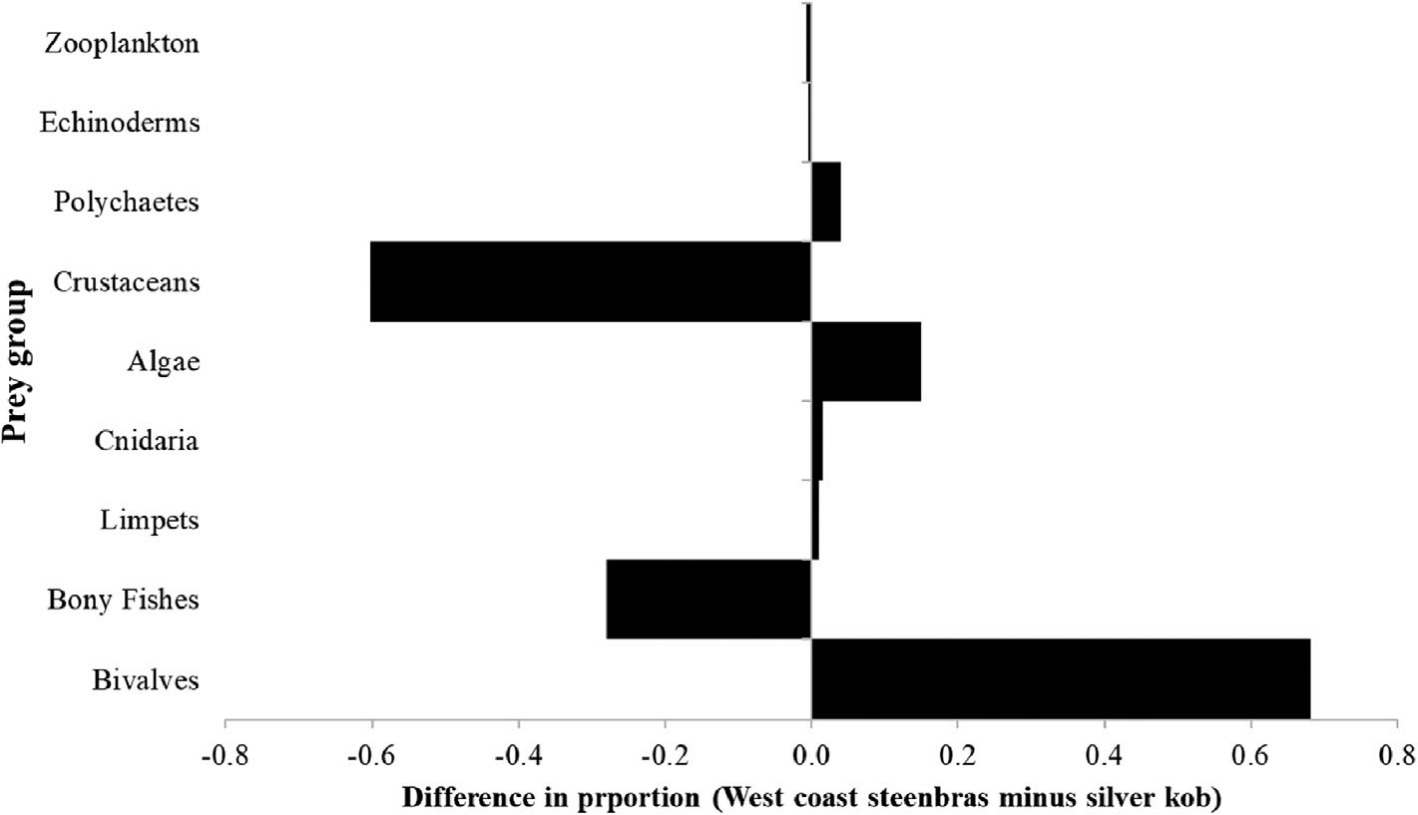
Difference in diet proportion between west coast steenbras *Lithognathus aureti* and silver kob *Argyrosomus inodorus* for niche overlap analysis based on Schoener's index.

There was a low dietary niche overlap of 0.11 (11%) between west coast steenbras and silver kob, obtained using the Schoener index. Bivalves are the main food source for west coast steenbras, with minimal overlap with silver kob, which consumes mostly crustaceans and bony fish. There was a moderate overlap for algae and echinoderms, whereas zooplankton consumption leans slightly toward silver kob (Figure 9).

## 4 DISCUSSION

This study, for the first time, compares the diet and estimates the niche overlap of west coast steenbras and silver kob at Meob Bay, Namibia. West coast steenbras exhibited generalist and omnivorous feeding behavior, with bivalves emerging as the most crucial prey item. Although silver kob is also omnivorous, crustaceans and bony fishes dominated its diet. Schoener's index suggests a relatively low overall niche overlap between the two species, emphasizing their distinct feeding habits and ability to coexist within the ecosystem.

In addition, the results indicate that the SFI of west coast steenbras was highest during the summer months and lowest during winter, which also corresponds with peaks in GSI, HSI, and CF. The highest SFI in January and October could be attributed to the abundance of prey items during these months, which may have led to increased feeding activity among both west coast steenbras and silver kob and therefore increased storage in both fat tissues (reflected by CF) and liver tissues (reflected by HSI). The low SFI in winter months could be due to reduced food availability and decreased feeding activity, as colder temperatures can limit the metabolism and digestive processes of fish.

The monthly variation in SFI observed in west coast steenbras could be related to temporary food availability at the time of sampling, because the sudden high feeding intensity in July (austral winter) was not reflected by their general body condition (CF) and liver index (HSI), which peaked in the beginning and at the end of the warm period (October and March–April). The apparent fluctuating and temporary food availability in winter is also reflected by the high proportion of empty stomachs and low SFI in June, sampled 1 month before in 2022 (Figure 3b). This points toward west coast steenbras being opportunistic feeders that feed in both summer and winter periods as soon as the food is available, but with probably generally higher food availability in spring and summer, and therefore increased condition in spring and summer, reflected by the peak upwelling periods in southern Namibia in spring and summer (Bartholomae & van der Plas, 2007). A more in‐depth seasonal study, considering factors such as prey availability and water temperature, would provide clarity on the variations in the SFI of west coast steenbras in southern Namibia.

It is said that feeding intensity is influenced by the reproductive cycle, as spawning activity can affect feeding behavior and energy allocation in fish (Ouakka et al., 2017). For west coast steenbras, the peak spawning activity takes place in spring and summer, with peaks in October and April (Shikongo, 2024) matching the peaks in condition period (present study). For silver kob, migration to the Meob Bay area for spawning occurs in summer (Kirchner & Holtzhausen, 2001). They appear to invest their energy in migration and spawning and also feed there during the spring and summer spawning period, as shown in the present study.

Apart from bivalves, other prey items of west coast steenbras included algae, bony fishes, polychaeta, crustaceans, cnidaria, and limpets, which were found in small quantities. Maritz (2020) found a similar diet in west coast steenbras inhabiting mining ponds in the Namdeb's southernmost licensed mining area, which extends approximately 100 km north from the Orange River mouth and contains multiple marine ponds created by mining activities. The diet of west coast steenbras in these mining ponds consisted of mussels, small fish, and crabs being their preferred food. Similarly, Holtzhausen and Kirchner (2001) observed that black mussels (*C. meridionalis*) and brown mussels (*P. perna*) were their preferred food in the southern population, Meob Bay, but more than half of the population's stomachs were empty, suggesting a lack of food availability during the period 1995–1999. In the present study, 16.3% of stomachs were empty (32 out of 196 west coast steenbras). Lucks (1970) also recorded that in Sandwich Harbour, west coast steenbras consumed amphipods, polychaetes, and *Mytilus*, possibly brown mussels, as the recorded arrival of *Mytilus galloprovincialis* (a bivalve) in southern Africa was after Lucks' observation in 1970 (Holtzhausen & Kirchner, 2001; Lucks, 1970; Maritz, 2020). The data on the feeding habits and diet composition of other species from different areas also indicated similar results. For example, the diet of the white steenbras (*Lithognathus lithognathus*) mostly consisted of bivalves, Polychaeta worms, crustaceans, mollusks, algae, mysids, and echinoderms (Bennett, 1993). Similarly, it was shown that the red steenbras (*Petrus rupestris*) and sand steenbras (*Lithognathus mormyrus*) diet also consisted of similar species (Smith & Smith, 1986).

All the size classes of west coast steenbras had a high IRI of bivalves in their diet, with the smallest size class (25–35 cm FL) having the highest percentage IRI. Fish in the size class 35–45 cm FL were omnivorous and preferred mostly bivalves followed by algae, whereas the fish in the size class 45–55 cm were mostly carnivorous, and their diet mainly consisted of bivalves with a presence of organic materials. The size class >55 cm FL mainly consisted of algae with the presence of organic material. This means that the west coast steenbras mostly prefers plant material, and as they grow larger, they might tend to either feed less frequently or have a higher metabolism. Stomach contents for another Sparidae, annular seabream, *Diplodus annularis*, indicate that, unlike juveniles, annular seabream adults are markedly carnivorous, and their diet is composed of annelids, mollusks, bivalves, crustaceans, and echinoderms (Chaouch et al., 2014). This is different in west coast steenbras in the present study, as they appear to occupy the same niche throughout from juvenile to adult fish.

All three sexes (female, male, and hermaphrodite) of west coast steenbras consisted of high percentages of bivalves within their diet. This species is a protandrous hermaphrodite; that is, it changes sex from male to female around the FLs of 46.0 to 48.5 cm (Shikongo, 2024). Based on the current results, west coast steenbras consumed algae at the size class of FL <45 cm, and therefore, it was also classified as an omnivore at that length class, which is similar to the length at which it begins to change sex. There was no significant seasonal variation in the diet of west coast steenbras, which again confirms them as opportunistic feeders that make use of temporary and sporadic increases in food availability. A similar diet study was done on the sand steenbras (*L. mormyrus*) by Santic et al. (2010), who also found low seasonal variation in the diet of this species in the Adriatic Sea, where bivalves dominated the diet composition throughout the year.

Silver kob exhibited a diverse diet, including crustaceans, bony fishes, bivalves, zooplankton, algae, and echinoderms. Thus, this species is omnivorous but prefers carnivorous prey as adults, with the diet of adult fish consisting predominantly of crustaceans and bony fishes. Crustaceans (shrimps and/or prawns) were the most important prey item across all size classes, sexes, and seasons. A study on southern meagre (*Argyrosomus hololepidotus*) in Australia also indicated that their most important prey items were fish, followed by a subphylum of crustaceans and bivalves (Shekari & Hashemi, 2012). Similarly, mulloway *A. japoonicus* in Australian waters feeds on small fishes and shrimps (Silberschneider & Gray, 2008) and in Khuzestan coastal waters (Iran) feeds on fishes and invertebrates (Shekari & Hashemi, 2012).

The smaller silver kob fed mostly on crustaceans, particularly shrimps, whereas the larger silver kob fed mostly on bony fishes, specifically the *Clupidea* spp. The medium‐sized fish fed on varying prey items. Silberschneider and Gray (2008) also found a predominance of fish in adult mulloway *Argyrosomus japonicus* diets. Previous research on silver kob has shown that small crustaceans, especially penaeids and mysids, dominated the prey of small silver kob (20–30 cm TL). Mysids were also found in the stomachs of *A. hololepidotus* as large as 65.6 cm (Smale & Buxton, 1989). In the present study, there were no significant differences between sexes in relation to the major prey groups in the diet of silver kob. Only some prey items, such as bivalves, algae, and echinoderms, were not consumed by females, but there were high diet overlaps between sexes regarding the major prey groups.

Silver kob were mainly captured in summer in the study area and may show some seasonal variation in diet as they move between areas. Seasonal variations in the food variety were found in the stomachs of other Sciaenidae by previous studies. For example, Smale and Buxton (1989) found that crustaceans, mainly prawns, were more abundant in the diet of southern meagre during summer, contributing up to 70% of the diet. Babatunde et al. (2020) found seasonal diet variation in cobia *Rachycentron canadum* related to the seasonal variation in prey abundance in the same habitat.

The low Schoener index value (0.11) in the present study confirms that west coast steenbras and silver kob are exploiting different food resources. This is supported by differences in their feeding behavior, habitat preferences, and specific adaptations to different types of prey. Studies have shown that closely related species occupying similar habitats can partition their resources to reduce competition for limited food resources (Schoener, 1974). With low niche overlap, there is likely to be reduced direct competition for food resources between the two species. This contributes to their successful coexistence with a low competitive exclusion, allowing both species to persist in the same ecological community. This observation aligns with the principle of niche differentiation, which proposes that species occupying similar niches will evolve to minimize competition and maximize coexistence (MacArthur & Levins, 1967). Niche differentiation, feeding specialization, and habitat partitioning of sympatric species support findings from other studies, for example, four sympatric cryptobenthic reef fishes in the Caribbean (Brandl et al., 2020) and three tuna species in the Solomon Islands (Jiang et al., 2022). Not in all sympatric species, niche differentiation and/or habitat specialization is evident. For example, Abdul‐Razak and Al‐Hassani (2023) evidenced a high similarity (0.75) in diet between a Sparid and Sciaenid fish species in the Arabian Gulf (the same families as west coast steenbras and silver kob, respectively), both species mainly feeding on fishes and crustaceans.

Understanding niche overlap is crucial for comprehending the broader ecological interactions within an ecosystem. The low overlap between west coast steenbras and silver kob in the present study suggests that each species may have a unique role in the trophic dynamics of their environment. Such insights are important for predicting how changes in one species' population or behavior might affect others within the ecosystem.

Knowledge of the dietary preferences and niche partitioning of west coast steenbras and silver kob can inform strategies for sustainable management of the mixed fisheries of these fish populations when considering multispecies models. Additionally, understanding the ecological roles of these species aids in making informed decisions regarding protected areas and habitat conservation (Jackson et al., 2001). The results prompt further questions that could be addressed in future research. Investigating the specific factors driving the low niche overlap, such as environmental variables or biological interactions, could provide deeper insights into the ecological dynamics of these species. Niche differentiation can be a positive adaptation that allows temperate and tropic species to co‐occur in range expansion under changing climate (Kingsbury et al., 2020) and is therefore likely to benefit both silver kob and west coast steenbras. Additionally, studies on the potential impacts of anthropogenic activities on their feeding ecology and resource availability would be valuable for conservation efforts.

The food preference of west coast steenbras and silver kob differs at this location and at their preferred habitat, reflecting the adaptable nature of the feeding behavior and habitat preferences of the members of the two families. That is, west coast steenbras are found on sandy bottoms from shore to 10 m depth, and juveniles are found in the surf zone; they also inhabit rocky areas (Bianchi et al., 1999). Because bivalves are mostly found in abundance within these habitats, their diet mainly consists of a high number of bivalves. On the contrary, silver kob inhabit sandy shores at around 20 m depth or low‐profile reefs and rarely enter the surf zone. Within this habitat, the shrimps and small pelagic fishes are abundant (Bianchi et al., 1999), which explains the high abundance of these prey items in the diet of silver kob. These findings support the hypothesis of microhabitat‐driven dietary specialization within the same environment. However, silver kob exhibits greater habitat coupling than west coast steenbras, which is further reason for west coast steenbras' vulnerability to overfishing and environmental changes in this near‐threatened species (Mann et al., 2014). Quantifying prey availability in relation to dietary abundance within these habitats would provide further insights into this hypothesis.

In terms of feeding behavior, west coast steenbras are active predators that hunt during the day and feed on a variety of invertebrates and small fish. Silver kob, on the contrary, are more generalist and tend to feed on smaller prey items, such as crustaceans and small fishes, at night. This difference in feeding behavior also helps to minimize competition for resources. Furthermore, the two species have different behavioral patterns. West coast steenbras, similar to their close relative the red steenbras (*P. rupestris*), tend to be more solitary and territorial (Brouwer, 2002), whereas silver kob form schools and are more social (Kirchner, 1998). This means they are less likely to come into contact with each other, further reducing the potential for competition for food.

In conclusion, in the present study, we demonstrated diet and habitat partitioning of the two sympatric species, silver kob and west coast steenbras, in southern Namibia. These adaptations have allowed these two most abundant surf zone fishes in Namibia to coevolve and coexist successfully. In addition, we highlighted that west coast steenbras are more vulnerable than silver kob to climate change or range expansion, as they are less likely to couple habitats.

Therefore, we recommend to investigate and contrast the dietary makeup of southern and northern stocks of west coast steenbras, as well as silver kob caught in northern and southern Namibia, to understand how this impacts their ability to grow, reproduce, and survive in the different areas and habitats, as these areas are differentially affected by climate change.

## AUTHOR CONTRIBUTIONS

Arariky S. Shikongo: data acquisition, data analysis, and writing the first draft. Margit R. Wilhelm: conceptualization, data analysis, funding acquisition, supervision, and writing—review and editing.

## FUNDING INFORMATION

This research was funded by the Department of Fisheries and Ocean Science at the University of Namibia and the Oranjemund Angling Club (OAC).

## Supporting information


Data S1.
