# Supplementary material for: Diet, feeding, and niche overlap of west coast steenbras (Lithognathus aureti) and silver kob (Argyrosomus inodorus) in the northern Benguela
Source: J Fish Biol. 2024 Aug 23;105(6):1703–14. doi: 10.1111/jfb.15914 (PMC11650930; doi:10.1111/jfb.15914)
Supplement: Supplementary file 1 — Data S1. [file JFB-105-1703-s001.docx]

# Diet, feeding and niche overlap of west coast steenbras (*Lithognathus aureti*) and silver kob (*Argyrosomus inodorus*) in the northern Benguela

Arariky S. Shikongo and Margit R. Wilhelm

**Supplementary information**


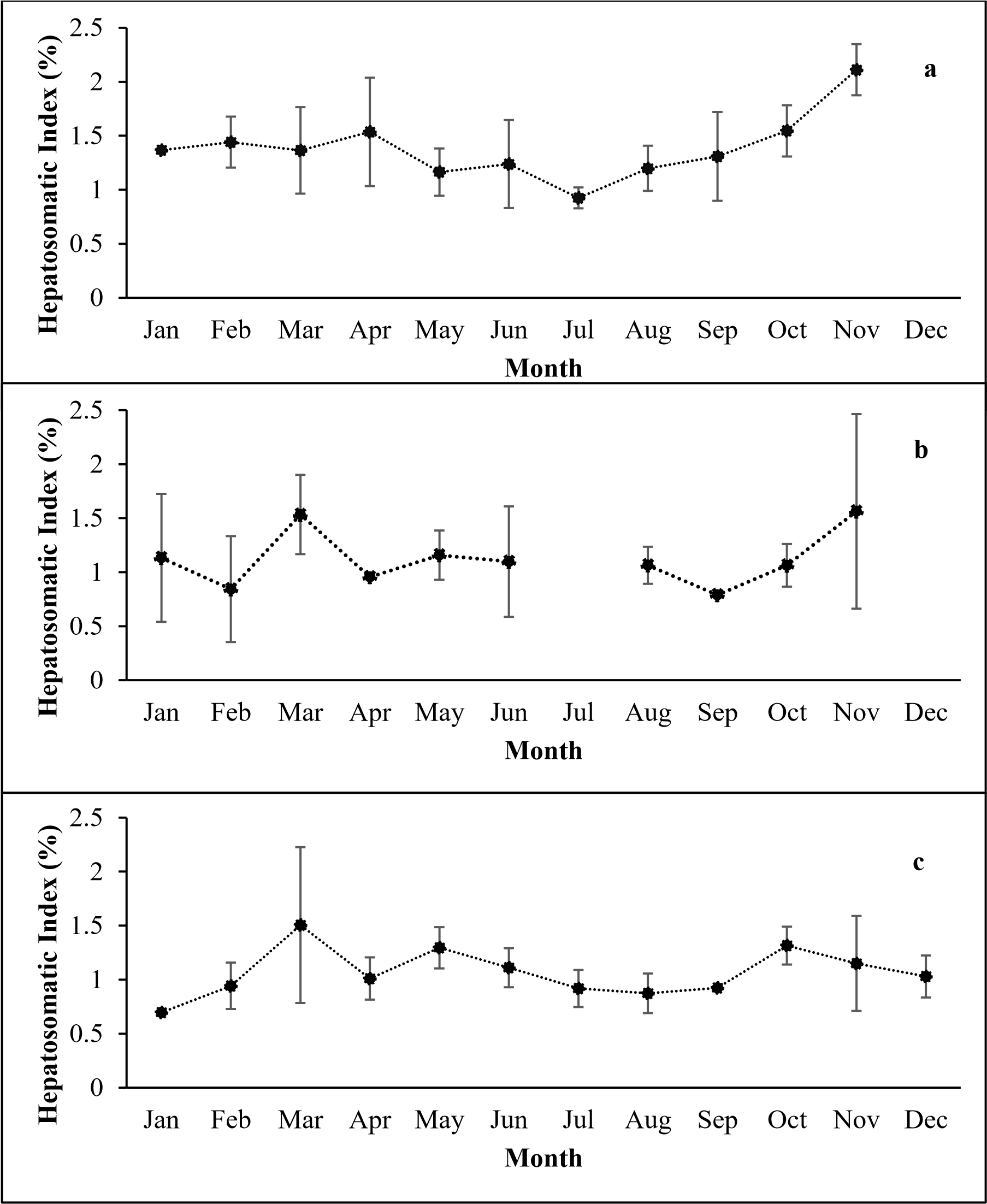


**Figure S1.** Monthly variation in the mean of the hepatosomatic index (HSI) ± standard deviation of female (a), male (b), and hermaphrodite (c), West coast steenbras from Meob Bay, southern Namibia, (2020-2022).

**Figure S2.** Monthly changes of mean condition factor ± standard deviation of female (a), male (b), and hermaphrodite (c), West coast steenbras from Meob Bay, southern Namibia (2020-2022).
